# Remarks upon the Law of England as It Relutes to the Crime of Fœticide

**Published:** 1832-09

**Authors:** Alfred S. Taylor

**Affiliations:** Lecturer on Medical Jurisprudence at Guy's Hospital.


### MEDICAL JURISPRUDENCE.

*Remarks upon the Law of England as it relutes to the Crime of Fœticide.* By ALFRED S. TAYLOR, Lecturer on Medical Jurisprudence at Guy's Hospital.

MEDICAL Jurisprudence is now so rapidly gaining ground as a branch of professional study, that any apology for the appearance of this paper in a Journal, the avowed object of which is to aid in the diffusion of every science, directly or indirectly connected with medicine, will perhaps be deemed superfluous.

There are probably few crimes against which human feeling is more prone to revolt, than that which involves the wilful destruction of the offspring in the womb, or, as is termed by our law, *abortus procuratus;* and, on this account, it would appear indispensable that the legislature of a country should not only carefully guard against its commission, but punish it with impartiality and certainty when it is once clearly proved to have been committed. How far these ends are accomplished by the present statute law of England, (9 Geo. IV. c. 31,) remains to be seen; and if the writer should differ from some great legal authorities in views relative to this interesting question, he trusts that he may fairly justify himself by appealing to the improved state of science and civilization, which the revolution of a few years has so happily brought about.

The main objection to the law, as it at present stands, appears to him to be as follows:

That, in principle, it involves a distinction between offences strictly similar, (and therefore a difference in the degree of punishment awarded,) which is not only at variance with the sound maxims of physiology upon which it is assumed to be built, but directly opposed to every feeling of justice and humanity.

Previously to making any comments upon the actual law, it will be worth while to inquire whence the principle upon which this distinction is founded, originated; and to trace the various modifications which it has undergone up to the present period of time. No individual can look back to the codes of antiquity without failing to remark in how many instances the institutions which they contained were either directly founded on, or supported by the approved physiological and medical hypothesis of the day. The opinions of HIPPOCRATES, of ARISTOTLE, and of GALEN, had, deservedly, their influence with the philosophic legislators of antiquity; and, although the lapse of centuries has fully exposed the fallacies into which these great teachers of mankind were led, through the adoption of an erroneous system of philosophy; although their hypotheses have, for the most part, vanished before the light of experience, yet we cannot but applaud the spirit with which physicians and jurists were animated to render the criminal jurisprudence of those times as perfect as the nakedness of their resources would allow. But what are we to say of the legislature of modern Europe, when we find that those very hypotheses which are now regarded by physiologists in their true light, namely, as the idle and vain speculations of those who lived in the infancy of science; that these, I repeat, are still to be found incorporated with the statute laws of its various countries, and are made to serve as standard rules for the prevention and punishment of crimes which involve the destruction of life? Is it not, indeed, a reflection upon the common sense of mankind, and a libel upon the present state of science, that legislators should so far neglect the high trust reposed in them as to frame laws upon hypotheses which would otherwise be mouldering in oblivion?

It was a favorite opinion with the ancient Stoics, that the soul did not unite with the body until the act of respiration was accomplished, and consequently that the fœtus in uteri could not be considered to possess a living or rational soul! The early Roman legislators, guided and influenced by this sentiment, did not look upon the procuring of abortion as a crime; the fœtus, until the period of delivery, being designated as *pars viscerum matris;* a term which is still preserved in the law of Scotland, although under a more restricted signification. Such a principle being allowed to run its course, gave birth, as might naturally be expected, to a gross corruption of public morals, which remained unchecked until the reigns of the Emperors Severus and Antonine. It was about this period that the sect of Zeno began to give way to that of the Academicians; and now, for the first time, we find noticed that modification in the law relative to abortion wilfully procured, the substance of which is still preserved in our modern codes of jurisprudence. According to the views of these philosophers, there was *a period* at which the fœtus in utero became endowed with *animation,* when, in other words, it ceased to be, as the former law expressed it, "*portio viscerum matris;*" and now the writings of the fathers of medicine were consulted, in order that this period might be at once fixed and determined. It had been laid down by Hippocrates, that there was a difference in the period at which the fœtus became endowed with vitality, depending upon its *sex:* the male fœtus, according to his doctrine, acquiring this living principle on the thirtieth, and the female upon the fortysecond day after conception. GALEN, on the other hand, believed that organization did not commence until about the fortieth day after impregnation in fœtuses of either sex, and that it was not until this period that it could be said to acquire a living and rational soul! By others, again, a more vague distinction was made, in general terms, between the imperfect embryo and the perfectly formed fœtus; the destruction of the former being considered as a trivial offence, punishable by fine; of the latter, as a crime equal in enormity to homicide itself.

The introduction and subsequent diffusion of Christianity did not work that change which might have been expected, with regard to this unsettled state of legislation upon a crime that appears to have been so prevalent. It is true that the fathers of the church, and among these TERTULLIAN, strongly denounced it,* even when committed at the earliest period of gestation, or when the fœtus was in the state of embryo: and it was even determined by the council held at Constantinople in the year 692, that those convicted of the crime should, in *all cases*, be punished as murderers. The canon law, however, which prevailed in Rome, and in the western parts of the empire, still maintained and acted upon that distinction in the offence which I have above indicated; a distinction which, as FODERÉ truly remarks, is subversive of all morality, and worthy of the dark and unenlightened times in which it took its origin!

It was the main policy of the Romans, as well as of their immediate successors, to carry into every country those principles of jurisprudence which had for so long a period ruled the greater part of the then civilized world. Hence it was not to be expected that Britain would escape the effects of this deluge of municipal institutions; but although, doubtless, many of these became incorporated with the customs of the ancient Britons, and yet remain as part of our common law, it is impossible to distinguish them from those numerous other innovations subsequently made by the Picts, the Saxons, and the Danes. No mention of the crime, however, which I am considering, is to be found in those annals that refer to what may be called the military occupation of Britain by the Romans; and it is, therefore, probable, if indeed it existed, that there was no definite punishment assigned to it until the introduction of the great body of the canon law, in the reign

* Homicidii festinatio est prohibere nasci: nec refert, natam, quis eripiat, animam, aut nascentem disturbet. Homo est et qui futurus est.—*Tertulliani Apol.* ch. ix.

of the usurper Stephen. Rome had then passed, by a wonderful succession of changes, from a military domination under that of the professors of a religion, whose ambitious encroachments upon the civil policy and government of nations gave them, finally, the means of holding for centuries the whole moral world in a state of slavish subjection? The civil and the canon laws then began to spread over the greater part of the continent of Europe; and it is not a little remarkable that they have to a great degree preserved their influence, even up to the present enlightened period, in almost every country belonging to this quarter of the globe, with the exception of our own.

The first notice of "*abortus procuratus*" as a crime in the laws of England, and the first certain indications which we have of a punishment being attached to it as such, must be dated from this period, namely, from about the reign of Stephen; for Bracton, one of our oldest legal authorities, who lived and wrote in the reign of Henry III., says, in allusion to it, "Si aliquis mulierem pregnantem percusserit, vel ei venenum dederit, per quod fecerit abortivam: si puerperium jam formatum fuerit, et maxime si fuerit animatum, facit homicidium." (*Bracton*, lib. iii. cap. 21.) Here, then, even by this old writer, we have the offence distinctly defined; but we may readily perceive that it is founded on precisely the same principle as that recognized by the canon law of Rome. We will now see what the great modern interpreter of the common law says relative to this offence. In the first Book of his Commentaries,* when speaking of the right of personal security, he expresses himself as follows: "Life is the immediate gift of God, a right inherent by nature in every individual, and it begins, in *contemplation of law*, as soon as an infant is able to *stir* in its mother's womb: for," continues he, "if a woman is quick with child, and by a potion, or otherwise, killeth it in her womb, or if any one beat her, whereby the child dieth in her body, and she is delivered of a dead child, this, though not murder, was by the ancient law homicide,† or manslaughter. But the modern law doth not look upon the offence in quite so atrocious a light, but merely as a heinous misdemeanour." Since the time of Blackstone, the legislature has twice interfered to specify more clearly the offence and its punishment. By the 43 Geo. III. c. 58, it was enacted that the attempt to procure abortion in a woman not being

* Blackstone's Comment. vol. i. p. 129.

† Professor Christison properly observes, that this distinction was formerly merely nominal, the punishment of murder and manslaughter being by the common law the same.

proved to be quick with child, should be deemed felony, the felon to be punished with fine, imprisonment, or transportation. If, however, it were proved that the woman had quickened, it should be deemed felony without benefit of clergy; and that, therefore, the individual should suffer capitally, as in the case of murder. By a most unpardonable omission of words in the more penal clause of this section of the statute, the intentions of the legislature were defeated; and although a man might suffer death for administering a drug to procure abortion, yet he might commit the offence with impunity if he employed mechanical means, which, as those who were instrumental in bringing forward this statute ought to have known, are far more efficacious and certain in producing the desired result than the administration of all the drugs which have been so highly extolled for this purpose. Upon a trial which occurred at Bury in 1808, under this section of the statute, the prisoners were proved to have attempted, but in vain, to produce abortion by drugs, and to have only finally succeeded in their purpose by the employment of instruments; yet, as the clause stood, the employment of manual violence could only be admitted to prove the *design* of the prisoners, and not the *fact* of their criminality. In order to amend this, as well as other defects which the operation of the law soon brought to light, the Act of Lord Ellenborough was repealed by that of Lord Lansdowne (9 Geo. IV. c. 31.;) and it is upon the provision of this statute that the prevention and punishment of the crime now depend.

The present law differs most remarkably from the ancient (at least, if we are to take the interpretation of Blackstone,) by not making it any offence for a woman to attempt that upon her own person, which it is felony in another for attempting upon her.* Thus, in the Lansdowne Act, it seems not to have been contemplated that a woman might, of her own free will, attempt to procure abortion without the advice or collusion of others; for the words of the statute specially limit the commission of the crime to those who *administer to the woman, or cause to be taken by her, any medicine, &c., or shall use any instrument with the intention of procuring the miscarriage.* So that, in this way, the offence may still be committed with impunity by the female herself, although another may be brought to suffer transportation, or even a severer punishment, by simply suggesting the means to her. By the ancient law

* It is worthy of remark, that the statement of Blackstone is not borne out by the quotation from Bracton, upon which he appears to have founded it. Bracton says, "Si *aliquis* mulierem," &c.; and the whole passage, in this respect, corresponds to the present provisions of the Lansdowne statute.

the *death* of the child expelled appears to have constituted the essence of the crime; for it expressly states, that if the child were killed in the womb, or if it were born alive and afterwards died in consequence of the means employed for its expulsion, it was considered homicide. Lord Lansdowne's Act, however, has no reference to this point, so that, whether the child survive its premature expulsion or not, the law would be equally violated. Lastly, with respect to the degree of punishment inflicted: by the ancient law, if the child died, whether before or after expulsion, the crime, as I just now stated, was deemed to amount to homicide; but this being, in all its forms, within the benefit of clergy, it is probable that the greater number of offenders escaped with a slight punishment. From Lord Coke's time* until the passing of the Ellenborough Act, "*abortus procuratus*" appears to have been only regarded as a heinous misdemeanour, and to have been punished accordingly. By the present law, the crime is in all cases held to amount to felony, but a most extraordinary distinction has been introduced relative to the degree of punishment, which is made to depend solely upon the fact of the woman having quickened or not: and it is to the nature of this distinction, more particularly, that I wish to call the attention of the reader.

The vague and absurd speculations of the Stoics and Academicians regarding the period at which the fœtus received animation, have already been noticed; and, by referring to Bracton, it will at once be seen that the old English law was modelled after the hypothesis of the latter;† for he says: "si puerperium jam formatum fuerit, et maxime si fuerit animatum, facit homicidium;" but he does not fix the period at which such animation was considered to take place. Blackstone, in treating of this point, says, "Life begins, *in contemplation of law,* as soon as an infant is enabled to *stir* in the mother's womb." This mode of expression evidently refers to the period of quickening; and it is this which the Ellenborough and the Lansdowne Acts have assumed as the barrier between the punishment of imprisonment and death for the commission of the *same* offence: I say the *same* offence; for, as I hope in the sequel to prove the distinction thus attempted to be established is arbitrary, in every sense of the word!

If, in this matter, we were to follow the language of the law

* 3 Ins. 10. Blackstone's Comment., vol. i. p. 129.

† "Non est homicidu, quæ abortum procurat, antequam anima corporis, sit infusa."—*Leg. Canon.*

or popular opinion, which in this, as in many other similar errors, is enlisted on its side, we might believe that the fœtus did not really become animated until the period of quickening: but what is the fact? Is there any thing which can for a moment lead us to entertain a doubt upon the subject? or, rather, would it not be to shock the good sense of my reader to attempt to prove that the process of quickening has nothing whatever to do with the infusion or incorporation of an animating principle? and how, therefore, are we to justify the application of so gross a fallacy to the *punishment* of a crime in any system of jurisprudence of the nineteenth century? It might serve to amuse us, like that institution said to have been framed in the time of Dagobert, by which an infant was not presumed to have survived its birth, for the purposes of inheritance, unless it had lived to see the four walls of the room in which it was born; but, surely, it is not fit to be treated in a light so serious as to constitute a line of demarcation between a transportable and capital felony!

In order to place this matter in a still clearer point of view, it will be necessary to make a few observations upon the so-called process of *quickening*, and the proofs by which it may be established. The word itself is well calculated to mislead the uninformed inquirer, and no doubt had its origin when the hypothesis, which I am now combating, was first received and adopted by physiologists. Still, as it has acquired a certain value by custom, it has not been expunged from the modern vocabulary of medical terms! although there is, doubtless, no individual in the profession who attaches any importance to its signification, or who believes for a moment that the sense in which it is used by the law is correct.

Much variety of sentiment has existed among writers, regarding the cause of this singular attendant on the uterine changes in the pregnant female. It has been usually ascribed to the first motions of the child which are perceived by the mother. But this explanation is insufficient; for the sensation of quickening is known to be altogether different from that which is experienced in any of its subsequent motions; and this, together with the circumstance of its occurrence about a certain period of gestation, would rather lead us to infer that it depends upon a change of position in the uterus itself, and not upon its contents; an inference which is still further borne out by the fact of this same sensation having been perceived in some forms of disease, where the uterus had become so large as to rise out of the pelvis into the abdomen. Whatever opinion we may adopt concerning the true cause of quickening, is altogether immaterial. Physiologists now seem generally to incline to the supposition that it depends upon a change of position in the uterus; and although this view of the case is not altogether free from objection, it may be regarded as the best which has been offered. On the other hand, it is universally acknowledged, (and this is the point to which the substance of my argument is chiefly directed,) that this symptom of pregnancy, if so it may be called, does not proceed from the incorporation of any living principle with the contents of the uterus, as is inferred and acted on by the law. Hence it remains to be explained how so absurd a fiction should be tolerated; even allowing the symptom to be constant, invariable, certain as to time, and easy of proof; all of which conditions are, as I shall now proceed to shew, absolutely wanting.

The sensation of quickening, so far from being constant and invariable, in some cases has not even been experienced at all; and, again, instances have occurred where the female has believed that she had quickened, although it has subsequently appeared that she was not pregnant. Now with respect to the *time* at which this symptom manifests itself, there exists equal, if not greater, uncertainty. From a comparison of the observations of ancient and modern writers, we may fairly presume that this sensation is experienced at some period between the tenth and twenty-sixth week, but that it most commonly happens about the sixteenth week after conception.* Is it possible, then, admitting such a statement, to connect the sensation of quickening with the absolute developement of the fœtus? Is it possible to conceive that the fœtus of a female who quickens at the twelfth week, is, physiologically speaking, so perfectly developed as that of another who does not quicken until the twenty-fourth or twenty-fifth week of gestation? And if we answer these questions in the negative, as undoubtedly we must, what are we to think of the reason assigned by the law, or of the dispensation of punishment under a statute, which makes an offence to vary in *degree* according to the presence or absence of so equivocal a sign of the pregnant state? Let us, however, take a practical illustration. A man may be tried for "abortus procuratus" on a female who has passed the *twentieth* week of her pregnancy, but who has *not* quickened: in such a case, the prisoner cannot be convicted capitally. Another may be tried for the offence upon a female who is only in the *thirteenth* week of her pregnancy, but who, through peculiarity

* Vide Medical Evidence in the Gardner Peerage Cause, by R. LYALL, M.D. (passim.)

of constitution, may have experienced the sensation which is required by the law, and in this case the prisoner may be capitally convicted and executed. Now, in these instances we have a difference of two months in the degree of development of the child, and yet he who has caused the expulsion or destruction of that which is the least perfectly formed is made to suffer the extreme penalty of the law; while the more atrocious offender is perhaps allowed to escape with a fortnight's imprisonment. Moreover, the feeling of quickening occupies so short a period of time, that an individual who commits the offence on the *evening* of the day on which it is perceived by the female, may forfeit his life; while, if he had attempted it in the *morning*, he might have escaped with a trivial punishment. It may be said that this is bringing the question to an extreme; but such is the *fact*, and such must be the effect of the law, as long as it is allowed to continue on the statute book. The application of such a principle to the punishment of any other crime would call for the loudest censure, and yet here it is tolerated and constantly acted up to. I do not mean hereby to assert that executions for this offence are frequent. It is well known that the trials of offenders under this statute are generally either upon the less penal clause, or, if a capital charge be preferred, the law is at once readily defeated by the bare assertion or deposition of the female.* This leads me to speak of the *mode of proof* tolerated by the law on so important a subject. In most cases the declaration of the female is received; and, to a physiologist, it would appear difficult how any other species of proof could be resorted to. But the law, in certain cases, where the female has a direct interest in pronouncing herself to be *quick with child*, empowers the judge to empannel a jury of matrons for the purpose of determining a question, which, if the female distinctly refuse to answer, can never amount to more than a conjecture. What physical proofs can there be to shew that a female has quickened, in the sense understood by the law? It is often difficult for the experienced accoucheur to determine whether she be pregnant or not; but here positive proofs of the presence of an incidental symptom of gestation are required in order to mete out the punishment of an offender! It is then no longer surprising that the capital charge should be so rarely established, when it must abso-

* By referring to the reports of trials for "abortus procuratus," which have taken place in this country since the passing of the Ellenborough Act, it will be seen that, although the female, in some cases, had passed the fourth month of her pregnancy, it has been almost uniformly maintained that she had not quickened, or felt the child alive within her; and the prisoner has thereby escaped the punishment awarded to his offence by the law.

lutely repose upon the bare ipse dixit of the female herself. Now, by the adoption of such a principle, it is quite manifest that the life of a prisoner is, in such cases, in the hands of the female; for if she choose to swear that she has quickened when she really has not, or vice versâ, it is a species of perjury which can never be proved against her, and which can only come to light by her own voluntary confession. This may likewise be considered an extreme view of the case; but no one can deny that a woman may be so far instigated by malice or revenge, as, where the law has placed it in her power, to swear away the life of one who perhaps may have brought her to disgrace and dishonour; and, on the other hand, her affection for the prisoner may, in some instances, lead her to declare falsely her condition. In either case the stream of justice will be polluted; a result which must always be looked for whenever the operation of the law is at variance with the common principles of equity.

From the preceding observations, then, we are, I think, fully justified in concluding,

1st. That the reason upon which the law founds a distinction between the major and minor offence of "abortus procuratus" is unsatisfactory; because it is built upon an assumption long since proved to be fallacious.

2d. That the sensation of quickening is merely an incidental symptom attendant upon utero-gestation, sometimes present, sometimes absent, and, in most cases, variable and uncertain as to the *time* of its occurrence.

3d. That, therefore, it is totally unfitted to form a line of demarcation between a capital and non-capital felony.

4th. That it is not to be established by physical proofs or by the evidence of others, but must rest entirely upon the declarations or depositions of the female, who herself may be deceived by mistaking the symptoms of other diseases for it, or may voluntarily misrepresent her condition.

5th. That, by the establishment of such a distinction between offences which differ not in *nature* or *degree*, but merely as to the *time* of their commission, the law opens a door to fraud and collusion by which the guilty may escape, and the innocent be brought to the scaffold.

I have said that these offences do not differ in *nature* or *degree*; and it appears to me that this has already been proved by the illustration offered in a preceding page, where I have said that a man may be convicted capitally for the offence committed in the thirteenth week of pregnancy, for which another, under *common circumstances*, would only be imprisoned or transported; a narrowness of the pelvis and a greater development of the uterus, constituting, probably, the sole difference between the pregnancies of the two females, and certainly giving rise to all the difference in the degree of punishment inflicted on the two prisoners.

It must surely appear to all to be in the highest degree unjustifiable to lay down a distinction in law respecting a natural phenomenon, where none can exist in nature. If it were a question which involved the right of personal liberty or the right of property, it might be sanctioned by the necessities of the state, and custom would give to it the authority of law, although, perhaps, the rule founded upon it might be somewhat harsh in its operation. Such a principle ought not, however, to be adopted, much less acted upon, where the life of a human being is at stake, the more especially where we find a fiction which originated in a time of darkness, allowed to prevail over that knowledge which a long course of centuries has obtained for us. It will hardly be maintained that this offence, when perpetrated at the third month of gestation, is less atrocious than when committed at the fourth or fifth month, or that this, again, is less in degree than when it is committed at the sixth or seventh month; for it must be evident, upon a slight reflection, that no line can be drawn: and, of all the attempts of this kind, that adopted by the legislature has been proved to be not only most open to objection, but absolutely dangerous in principle, because it places so great a trust in the hands of the female. I am aware that it has been argued by some physiologists, that we ought in these cases to make a distinction between the mature fœtus and the embryo; but no satisfactory reason has ever been assigned for this, nor has it been shewn to be in any degree necessary. If it could be proved that the life of the fœtus at the second month of gestation were less important to itself, or that it had smaller claims upon the protection afforded by the laws of society, than a fœtus advanced to the sixth month of pregnancy, it would be a step towards proving that such a distinction was justifiable. But it is forgotten that the law, as applied to the crime of infanticide, would be altogether at variance with such a principle as this, even allowing it to be established. By the same act, the destruction of the newly born infant and the murder of the first senator of the land, are classed as crimes of the *same dye*, are punished in the *same manner*, and are directed to be proved by the *same rules* of evidence. Now, in this case, we do not follow the degree of development in the being as the rule or standard for the infliction of punishment; and how, therefore, are we to reconcile the application of such a rule to the criminal destruction of the unborn child?

There is another ground upon which this distinction in the crime of "abortus procuratus" is objectionable, and in which the law appears to be at variance with itself. An infant in *ventre sa mere*, or in the mother's womb, is supposed in law* to be born for many purposes, or, as it is expressed by the civil code, *Qui in utero sunt ex jure civili intelliguntur in rerum naturâ esse cum de eorum comodo agatur.* Thus the child may take land by descent, may have a guardian assigned to it, and in executory devises it is considered as a life in being. Now, if we are to take these provisions in their strict and natural signification, we must consider that they apply to the child whether before or after quickening; and hence the life of the fœtus at the third or fourth month is as much bound to be respected by the laws of society as when it has reached to a more advanced period of pregnancy; and yet the Lansdowne statute treats the destruction of the former as an offence comparatively trivial, while the destruction of the latter is considered as tantamount to murder, and is punished with the utmost severity of the law!

As then, in a moral and physiological point of view, there can be no difference in the degree of the crime, and as the civil rights of a child in *ventre sa mere* must be equally violated whether the female has quickened or not, it is impossible to admit that there ought to exist the least difference in the punishment of the two offences. It is also more than doubtful whether the provisions of this Act have caused that diminution in the crime which was probably anticipated by those who were instrumental in framing it, since the direct effect of the fallacious distinction incorporated in it has been rather to encourage the offence at an early period of pregnancy, than to prevent its commission after quickening: and the numerous cases of acquittal on the capital charge, where, as I have already stated, the female had passed the period at which women *ordinarily quicken*, prove that the more penal clause may be with ease and safety evaded. It would appear even that the light punishment assigned to the minor offence has been the means, not only of making the crime more frequent, in diminishing its apparent enormity, but of laying the foundation for another more heinous charge against the prisoner: since it is a well known fact that substances of a deleterious nature are employed on these occasions, and often, through ignorance or wilfulness, in quantities sufficient to cause the death of the female without effecting the purpose intended.†

* Blackstone's Comment. i. 130.

† Few assizes pass without the occurrence of cases of this description. The

The conclusion at which I have arrived is, then, that there should be no difference in the degree of punishment for this offence, at whatever period of gestation it may be committed, provided the wilful and felonious intent be fully proved: and now it may be asked, whether capital punishment should be *in all cases* inflicted, or whether the offender should be subjected to transportation or imprisonment merely. I might at once solve this question by replying that the same principle which would justify the law in executing a prisoner for what is *now* considered to be the major offence, would become equally applicable to the minor. I apprehend that there can be no doubt entertained upon this point, except by that sect of enthusiasts who denounce the punishment of death even where the law of God* and of almost every human community has ordained it, that is to say, in the wilful destruction of a fellow being, who would wish to try the experiment of putting an end to the first principles of all society and government, and to extend that mercy to the murderer which was denied to the murdered. It is not, however, solely as means of expiation that the punishment of death should be inflicted; but because, as Blackstone observes, it is this which, in an offence of enormity, such as that of which we are considering, tends most to the security of mankind and to the preservation of the species.

Assuming, then, that a human tribunal is justified in ordaining that death should be punished with death, we have a right to apply this principle to the destruction of a being in the *early* as in the *later* stages of its existence. No boundary can be established, no line can be drawn, no principle recogtrial of Russell, at the last Lent assizes at Huntingdon, will perfectly explain my meaning: The prisoner procured and gave arsenic to a female pregnant by him, for the purpose of bringing about her miscarriage; it operated so violently as to destroy her life. Now, in this case, the female not having quickened, an indictment under Lord Lansdowne's Act would have led to temporary imprisonment or transportation only. But the law says, if, in an attempted felony of this kind, a life be destroyed, the original intent is lost sight of, and the offender becomes guilty of murder. (Blackstone's Comm. iv. p. 26, 192; Foster, 259; and 1 Hale P.C. 429.) Although this principle of the common law was strictly applicable to the case in question, yet the prisoner was indicted upon the novel charge of being an accessory to the crime of felo de se, which the fifteen judges have since declared to be *contrary to law*, and therefore have quashed the conviction under it. The protection afforded by laws, however wise, just, or humane, must be considered as nugatory, unless proper means be taken to enforce their application in every case: for if a criminal be allowed to escape, it will matter but little to the public, whether it be through the inefficiency of the law itself, or through the incompetency of those entrusted with the prosecution of offenders!

† Vide Genesis, ix. 6; Numbers, xxxv. 31.

nised, which tends to put a lower price upon human existence at one period of gestation than at another, when, by the laws of man, the rights of the individual are in theory equal; and by physiology we are taught that the two states of being bear the same relation to each other which the newly born child does to the adult: or, to use the language of PERCIVAL, "to extinguish the first spark of life is an offence of the same nature both against our Maker and society, as to destroy an infant, a child, or a man; these regular and successive stages of existence being the ordinances of God, subject alone to his divine control, and appointed, by sovereign wisdom and goodness, as the exclusive means of preserving the race and of multiplying the enjoyments of mankind."

Having then, I hope, established that there are substantial grounds for revising this section of the Lansdowne statute, I will conclude the paper by making a few remarks upon a topic indirectly connected with the subject of quickening: I allude to "pregnancy in bar of execution."

A pregnant female, capitally convicted, is allowed by the law of England to plead her pregnancy in bar of execution,* and she is, "*favorem prolis,*" respited, provided she has quickened, until the period of delivery; but if she have not quickened, then the plea will be of no avail. In such a case, it will be manifestly to the interest of the woman to misrepresent her condition; and hence the judge is directed by the law to select twelve *matrons* to inquire into the fact, and if they bring in their verdict *quick with child,* (for barely *with child,* unless it be *alive* in the womb, is *not* sufficient) execution shall be staid,† &c. The distinction thus made probably arose in a time of ignorance, from the uncertainty of the signs of pregnancy previous to the period of quickening; and hence we need not wonder that it should have been received and acted upon by our forefathers; although, as I have above hinted, it is doubtful whether it was tolerated by the Roman law. To prove that it is unjust in its operation, would now be only a work of supererogation; for the arguments already adduced in speaking of the subject of criminal abortion, will equally apply here. If a convicted female be pregnant, whether she

* This coincides with that humane principle in the laws of ancient Rome, which sets forth "quod, pregnantis mulieris damnatæ, pœna differatur, quoad pariat;" but these do not appear to admit that barbarous exception recognised by the present law of England.

† It has been aptly remarked, that the judge might with equal propriety be directed to summon a jury of twelve *infants*; for it is hardly possible to understand that a set of ignorant women should be able to settle a question which has often defied the experience of the most celebrated accoucheurs.

has quickened or not, she ought undoubtedly to experience that lenity which the codes of antiquity assigned to a person in her condition; for to carry the sentence of death into execution where she has not quickened, would be to punish the future offspring for the crime committed by its mother: and, if the child, with which she was pregnant, should have lands devised to it, or be the subject of an executory devise, it would be a manifest violation of justice to deprive it of those civil rights by cutting it off in the dawn of existence, for a crime in which it had no participation!

