# Erratum

**Published:** 1832-10

**Authors:** 


ERRATUM in the last Number.—P. 197, for "frolis," read "prolis."

